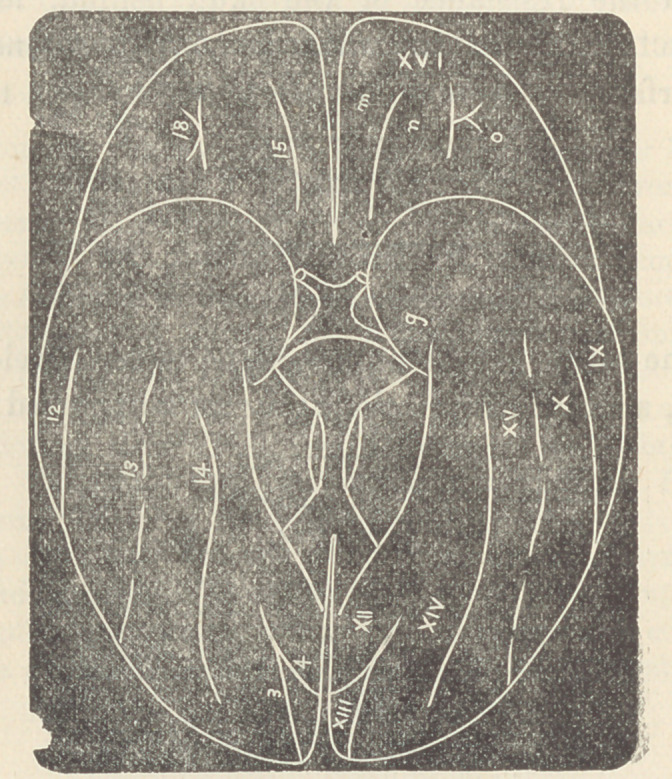# Cerebral Anatomy Simplified

**Published:** 1880-11

**Authors:** S. V. Clevenger


CHICAGO MEDICAL

JOURNAL & EXAMINER.

VOL. XLI.—NOVEMBER, 1880.—NO. 5.

### Original Communications.

ARTICLE I.

### CEREBRAL ANATOMY SIMPLIFIED. By S. V. CLEVENGER, M.D.

The human brain, like everything else in the universe, has been evolved from a simpler condition, and by a study of the forms through which the cerebrum has passed to its present complexity in man, we have been enabled not only to see the reasons for changes in appearances, but to simplify our methods of instructing students. I believe, with Prof. Burt G. Wilder, of Cornell University, that no medical student should be allowed to dissect the human cadaver until he has previously familiarized himself with the anatomy of the cat. If previous to this even, he should study the crayfish with Huxley's recent popular work on that crustacean as a guide, he will find that he not only has removed vast difficulties from his course of study, but will be able to continue through life an acquaintanceship with anatomical and physiological essentials, of which our present methods of teaching enable the average student to retain only a smattering, however industrious and earnest he may have been. In short, we cannot master human anatomy without a knowledge of comparative anatomy.

The primitive typical form of the mammalian cerebrum is as simple as this figure, an oblate spheroid :

In some fishes and birds it is globular, and therefore a still simpler form.

Rotate an ellipse about its shorter axis, and then cut the figure so produced into halves in the direction of its long axis, and the low form of the hemisphere from which we start is obtained. We have now a flattened surface which has been created by the two right and left lobes pressing against each other as the brain grew faster than the skull. In some fishes the original spherical appearance of the two lobes may be seen. The fissure which separates the two hemispheres is called the GREAT LONGITUDINAL FISSURE, and this cleft is along the inner flattened faces of the hemispheres, each of which surfaces is known as the MEDIAN SURFACE. The EXTERNAL SURFACE is the outermost and uppermost part. The BASILAR SURFACE is simply the lowest or under part of the cerebrum.

Draw two parallel lines lengthwise upon the external surface. These lines are sulci or little fissures produced by the folding in of the soft brain tissue as it develops more rapidly than the bones of the skull expand :

One such furrow may be seen on the hedgehog's cerebrum, the other is added in higher animals. But at the same time there appears another lobe growing in front of this; it is the frontal. The smallest frontal lobe, speaking relatively, is owned by the kangaroo, and adhering to our schematic representation is shown in the above cut. In the horse we find that these two lobes have crowded together, the frontal having grown much larger, but the original line of junction between the two is still evident in a fissure which in the elephant, monkey and man is called the SULCUS OF ROLANDO:

This frontal lobe or subsequent growth also has the two longitudinal cracks in the highest mammalia. But the skull still obstinately maintains its rigidity, lifting a little on top as we pass up the scale of animals. The hemispheres press back over the cerebellum only at this stage of high development; previously the cerebellum was uncovered by the cerebrum, and now another change begins. As the frontal lobe continues to grow, it crowds the occipital part back, and the latter cannot extend in the same direction any longer, but finds room below in the posterior part of the skull, whereupon this appearance is presented:

Where the posterior lobe folds under, the temporal lobe is forming, and the three great divisions of the cerebrum are more evident—the FRONTAL, OCCIPITAL and TEMPORAL LOBES. The first two are separated by the sulcus of Rolando, the last-named from the occipital by the large fissure, created by this folding-under process—the FISSURE OF SYLVIUS. The elephant's brain exhibits just this stage of development, and it is also to be seen in the human embryo.

In accomplished development the scheme of the cerebral fissures and sulci of man would be thus represented:

The temporal lobe passing forward as the olfactory lobe of quadrupeds diminishes in size and makes room for it.

But by gradual filling in of certain portions of sulci, breaks are made in their continuity and the system of folds is rendered complex. The original derivation, however, is not completely masked, for we may still trace the primitive furrows into the fully developed cerebrum of man.

The convolutions marked I, II, III, correspond in the frontal lobe to those marked X, IX, VIII. The upper posterior convolution VI being a continuation of X, while the point c of the fissure 11 has been pushed back by filling in of the space anterior to it.

The deeper furrows are known as fissures, and those less deep or constant as sulci. The numbers and letters in the following list correspond with those of the figure above:

CONVOLUTIONS OF THE EXTERNAL SURFACE.—I. Superior Frontal. II. Middle Frontal. III. Inferior Frontal. IV. Ascending Frontal. V. Ascending Parietal. VI. Superior Parietal. VII. Inferior Parietal. VIII. Superior Temporal. IX. Middle Temporal. X. Inferior Temporal.

The fissures and sulci of the external surface are: 2. Fissure of Sylvius. 6. Sulcus of Rolando. 7. Parietal Sulcus. 8. Præcentral Sulcus. 9. Superior Frontal Sulcus. 10. Inferior Frontal Sulcus. 11. Superior Temporal Sulcus. 12. Middle Temporal Sulcus. (Prof. Pansch, of Kiel, calls this the Inferior by discrediting the existence of the Inferior of other authors.) 17. Transverse Occipital Sulcus. 19. Inferior Longitudinal Occipital Sulcus.

Small parts of the convolutions conveniently designated Gyri are known as: a. the operculum (injury to which causes aphasia). b. Supra marginal gyrus. c. Angular gyrus (lesion of b and c causes blindness, etc). d. First occipital gyrus. e. Second occipital gyrus. f. Third occipital gyrus.

The median surface development proceeds in animal life as follows: With the advent of the frontal lobe, its inner face fuses with the occipital, or what becomes afterwards the parietal part of the occipital, on a line which divides the inner surface just as the sulcus of Rolando divided the external surface:

But the corpus callosum appeared in forms of life above the marsupials and prevented the backward extension of this crack. It had to pass backward over the corpus callosum thus:

And in this figure we see the corpus callosum (a broad band of fibres connecting opposite hemispheres, with the CALLOSO-MARGINAL SULCUS just above it.

As the hippocampus major was curled under and forward by the frontal lobe pressure, the rotation of it and the fornix, together with the resistance of the skull behind, folded in the PARIETO-OCCIPITAL FISSURE, which in monkeys extends across the external surface, but in man has been filled in upon that part:

When the calcar avis or hippocampus minor developed in the ape's brain, another fissure, the CALCARINE appeared:

The fully developed Median surface of the cerebrum is represented in this cut:

FISSURES AND SULCI.—6. Termination of Sulcus of Rolando. 16. Calloso-marginal Sulcus. 3. Occipito-parietal Sulcus. 4. Calcarine Fissure. 5. Hippocampal Fissure. 14. Collateral Sulcus.

CONVOLUTIONS AND GYRI.—XI. Marginal Convolution. XII. Fornix Convolution. XIII. Cuneus Convolution. XIV. Median-Occipito-Temporal Convolution. g. Uncinate (or hook) gyrus. h. Dentate gyrus. j. Paracentral gyrus (lesion here always causes paralysis). k. Præcuneus or Quadrate lobule. l. Descending gyrus.

This last cut shows the convolutions and furrows on the Basilar Surface:

Some of these parts have been mentioned under the heads of the other surfaces, as they may appear upon two surfaces. Those most clearly basilar are :

FISSURES AND SULCI.—13. Inferior Temporal Sulcus (which is an extra fold in the human brain due to the same causes that created the original external furrows—want of cranial osseous expansion). 15. Olfactory Sulcus (produced by the olfactory lobe, which in man has dwindled to a mere tract.) 18. Orbital Sulcus (which lies directly over the orbit and is created by lateral and frontal pressure).

CONVOLUTIONS AND GYRI.—XV. Lateral Occipito-Temporal Convolution. XVI. Orbital Convolution. m. Gyrus Rectus (inwards from and along olfactory sulcus). n. Middle Orbital Gyrus. o. Lateral Orbital Gyrus.

In addition to such fissures and sulci as are of constant appearance, a great number of sulculi or lesser furrows of an inconstant nature are also present, these of course cannot be enumerated. In young children and imbeciles the sulcus of Rolando will be found much farther toward the front than in fully developed brains. This is because the frontal lobe has suffered arrest of growth. This sulcus appears very close to the front in idiots and their retreating foreheads are cranial adaptations to this cerebral defect.

There are several expensive plaster models of the cerebrum on sale, one of the poorest of which retails for about ten dollars. Several much better models by German and French anatomists of note are also on the market, but their cost places them beyond the reach of the many. I have carefully examined the literature of the subject up to very recent dates, and by combining authoritative descriptions with a great number of casts I have made of brains from hospitals and insane asylums in Illinois, have recently had a large number of copies of my model made and placed on sale at two dollars each. They may be purchased of E. H. Sargent & Co., surgical instrument dealers, No. 125 State street, Chicago. The price fixed barely covers the expense of their manufacture. At the outset I had no intention of having these casts made, but, having been called upon for so many copies, I felt compelled to hand the model and its copying over to the medical supply store mentioned.

By means of this cheap cast, with this paper, and even as inaccurate a work as Gray's Anatomy, the student can in a few hours master much of the subject; but, as before remarked, it is far better to study nature direct, if possible. In the *Journal of*

*Nervous and Mental Disease* for October, 1879, April, 1880, and October, 1880, I have somewhat fully detailed my investigations and those of others. The articles referred to are respectively entitled "Cerebral Topography," "The Sulcus Rolando and Intelligence," and "The Plan of the Cerebro-Spinal Nervous System."

## Figures and Tables

**Figure f1:**
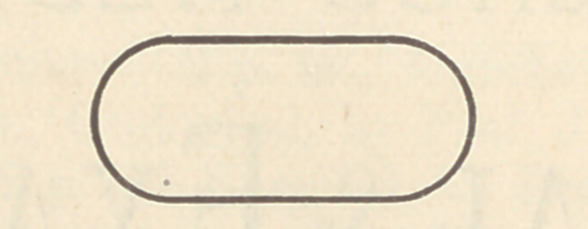


**Figure f2:**
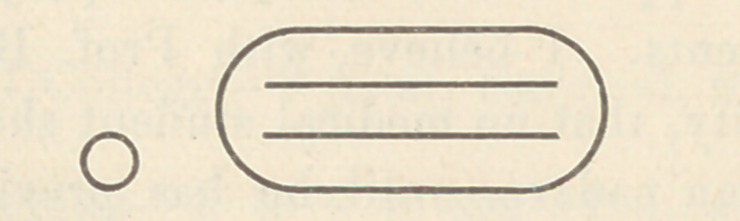


**Figure f3:**
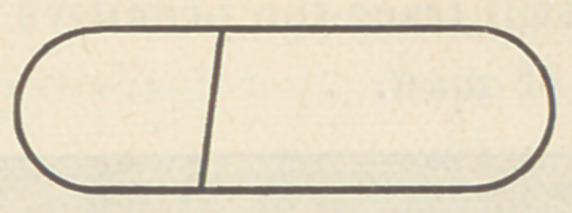


**Figure f4:**
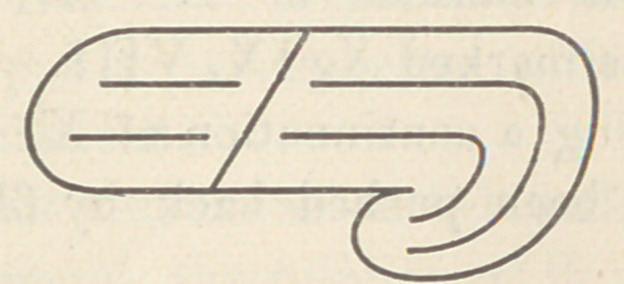


**Figure f5:**
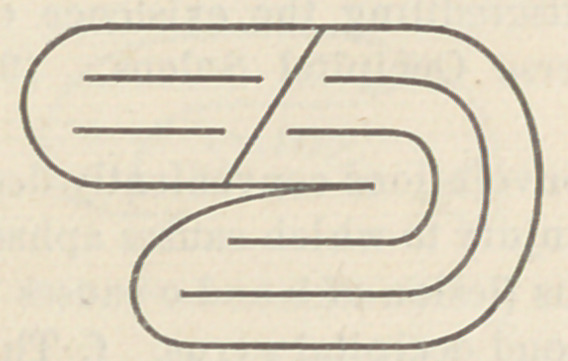


**Figure f6:**
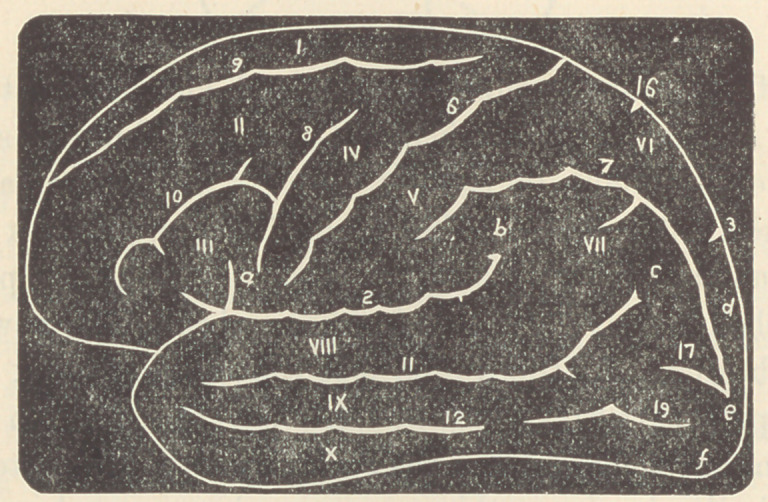


**Figure f7:**
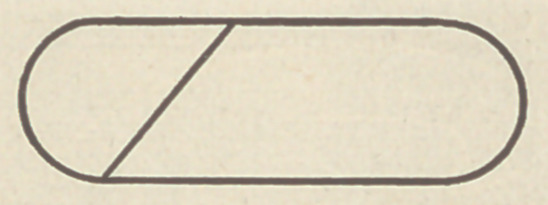


**Figure f8:**
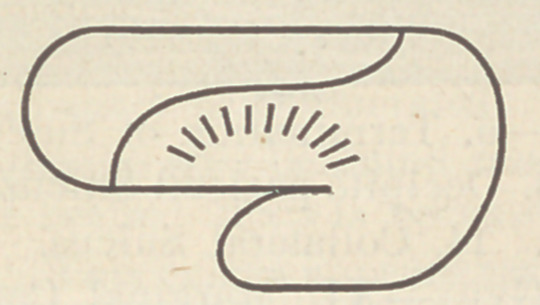


**Figure f9:**
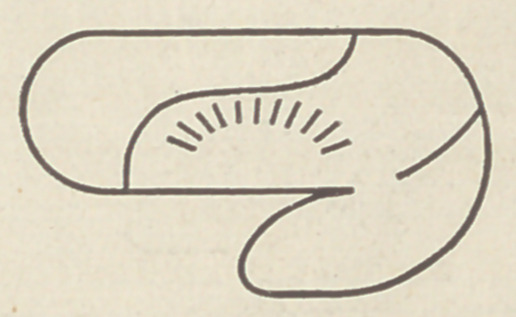


**Figure f10:**
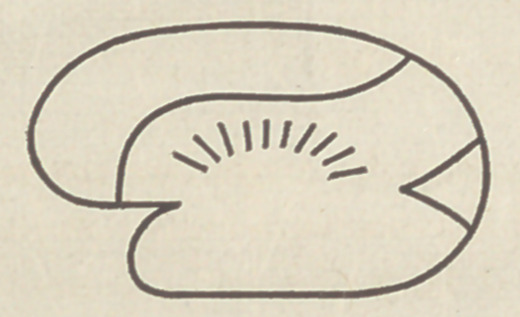


**Figure f11:**
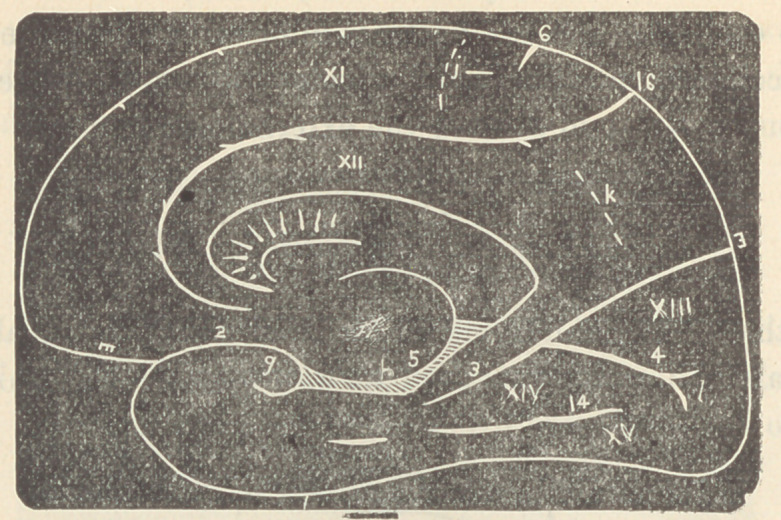


**Figure f12:**